# Iodine-125 radioactive particles antagonize hyperprogressive disease following immunotherapy: A case report

**DOI:** 10.1097/MD.0000000000022933

**Published:** 2020-10-30

**Authors:** Ning Yang, Pin-Liang Zhang, Zeng-Jun Liu

**Affiliations:** a Tumor Research and Therapy Center, Shandong Provincial Hospital Affiliated to Shandong First Medical University; b Internal Medicine Department, Shandong Cancer Hospital and Institute, Shandong First Medical University and Shandong Academy of Medical Sciences, Jinan, Shandong, China.

**Keywords:** iodine-125 radioactive particle implantation, Hyperprogressive disease, Immune checkpoint inhibitor

## Abstract

**Rationale:**

Increasing evidence has shown that immune checkpoint inhibitors are associated with hyperprogressive disease (HPD). HPD usually resulted in dramatically reduced survival duration, which limited the opportunity to administer other therapies.

**Patient concerns:**

A heavily pretreated lung adenocarcinoma patient experienced rapid progression of rib metastasis soon after immune checkpoint inhibitor -based combination therapy.

**Diagnoses:**

On the basis of radiographic and pathological findings, the patient was diagnosed with HPD.

**Interventions:**

We treated the patient with iodine-125 radioactive particle implantation to the metastatic lesions in the chest wall.

**Outcomes:**

The metastatic lesions shrank significantly 1 month later.

**Lessons:**

Early detection and adequate treatment are essential for prolonged survival when HPD occurs.

## 1 Introduction

Currently, immune checkpoint inhibitors (ICIs) have evolved as standard treatment modalities in advanced non-small cell lung cancer. They have also shown clear survival benefits as single-agent or combination therapy when compared with standard chemotherapy in treatment-naive or previously treated patients.^[1–7]^ However, increasing evidence has shown that these new immunotherapy drugs are associated with some novel tumor response patterns, such as delayed responses, pseudoprogressions, and hyperprogressive disease (HPD).^[8–9]^ Although the definition and incidence of HPD varied across studies, it always resulted in a dismal prognosis.^[10–13]^ However, the management of HPD has not been specifically addressed. Here, we present a heavily pretreated lung cancer patient who experienced HPD during ICI therapy and was successfully treated with iodine-125 (^125^I) radioactive particle implantation.

## 2 Case presentation

In August 2017, a 47-year-old nonsmoking Chinese man was referred to our hospital with a hard, immovable, and non-tender mass in the right supraclavicular fossa, approximately 2 × 2 cm in size. The patient had an Eastern Cooperative Oncology Group performance status score of 0. He reported no systemic disease. A contrast-enhanced total-body computed tomography (CT) scan (head to pelvis) revealed a mass and obstructive pneumonia in the upper lobe of the right lung. Along with this, lymphadenopathy (short axis > 15 mm) in the right upper mediastinum, supraclavicular fossa, and posterior cervical triangle and an osteolytic lesion in the right fifth rib were also seen. CT-guided biopsy of the lung mass revealed poorly differentiated adenocarcinoma. The adenocarcinoma cells were positive for CKpan, CK7, CD56 (focal), and CK5 (focal), but negative for TTF-1, CgA, Syn, P63, and P40. The positive expression rate of ki67 was 80% to 90%. Genomic analysis revealed no sensitizing mutations in the epidermal growth factor receptor gene or in the anaplastic lymphoma kinase gene.

Starting in September 2017, he received 3 lines of systemic chemotherapy before ICI treatment (paclitaxel and carboplatin plus bevacizumab for 6 cycles, followed by 4 cycles of bevacizumab monotherapy with partial response and a progression-free survival of 6 months, pemetrexed and cisplatin for 6 cycles with partial response and a progression free survival of 4 months, and anlotinib for 157 days with subsequent progressive disease). In addition, he also received brachytherapy with ^125^I radioactive particle implantation in the primary lung mass. The response evaluation criteria for solid tumors 1.1 was referred.

**Figure 1:**
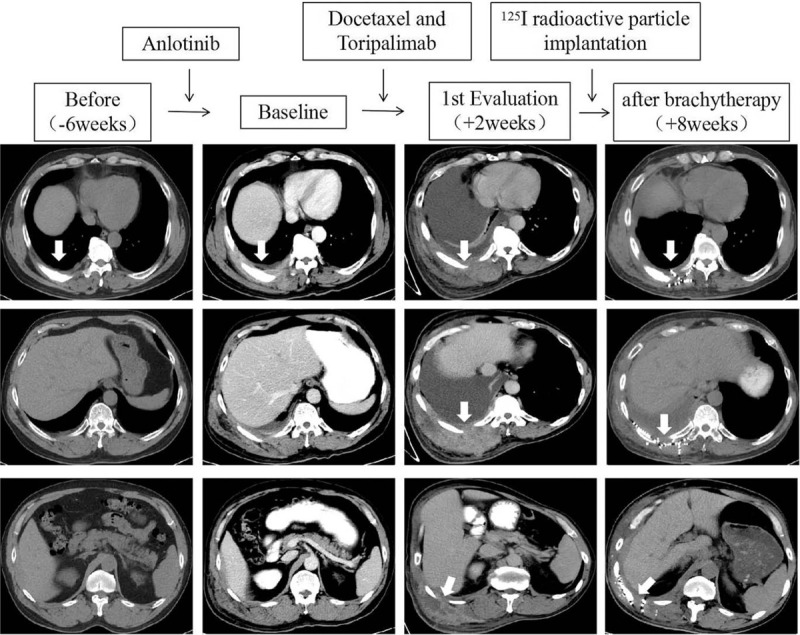
Scans before (−6 weeks), at baseline, at first evaluation (+2 weeks), and after brachytherapy (+8 weeks) in a heavily pretreated lung cancer patient who experienced hyperprogressive disease during immune checkpoint inhibitor therapy.

In March 2019, a contrast-enhanced total-body CT scan (head to pelvis) demonstrated partial response of the primary lung mass after 125I radioactive particle implantation, stable disease of the right fifth rib metastasis, and progression disease of the axillary lymph node metastases. Given the patient's performance status (Eastern Cooperative Oncology Group performance status 1), palliative treatment with docetaxel and toripalimab, the first domestic recombinant, humanized programmed death receptor-1 (PD-1) monoclonal antibody approved for use in refractory metastatic melanoma in China on December 17, 2018, was planned. Docetaxel and toripalimab were administered at 75 mg/m^2^ and 240 mg, respectively, every 3 weeks. After administration of the first dose, the patient began to experience a gradual worsening of his persistent right-sided chest pain. Two weeks later, a physical examination revealed a palpable right-sided chest wall mass. An enhanced CT scan showed progression of the rib metastasis. The metastatic tumor cells widely infiltrated the thoracic wall, invading the subcutaneous tissue (Fig. 1, 1st evaluation). Biopsy of the lesion revealed a poorly differentiated adenocarcinoma. Next-generation sequencing showed no targetable oncogenic alterations. Immunohistochemical analysis of programmed death-ligand 1 expression using the murine 22C-3 antibody revealed a tumor proportion score (TPS) of 0%. The primary lung tumor and metastatic lymph nodes remained stable. Immediately, the patient underwent CT-guided ^125^I radioactive particle implantation for the treatment of chest pain. Thereafter, the pain gradually subsided over the next weeks.

One month later, a CT scan confirmed that the metastatic lesions in the chest wall shrank significantly (Fig. 1, after brachytherapy). The patient then received systemic treatment with nanoparticle albumin-bound paclitaxel and gemcitabine every 4 weeks for 6 cycles. The disease has remained stable for more than 7 months until now.

## 3 Discussion and conclusion

Recently, several retrospective studies have described accelerated disease progression following treatment with ICIs, which was referred to as HPD.^[9–15]^ Although the calculated methods of HPD varied across studies, previous researchers consistently highlighted the importance of quantifying tumor growth speed to discriminate between natural course of tumor progression and progression due to ICIs.^[9–16]^ However, the evaluation of HPD in patients with immeasurable disease remains an open question.

In the present case, a sudden worsening of chest pain and a palpable chest wall mass, initially suspected to be hematoma due to rib pathological fracture or pseudoprogression, were observed. However, the following biopsy findings of poorly differentiated adenocarcinoma confirmed the diagnosis of hyperprogression, presenting as extensive tumor infiltration in the thoracic wall. The current HPD criteria are insufficient to perfectly evaluate the growth speed of non-measurable lesions. However, in the present case, the tumor growth speed accelerated suddenly and greatly after PD-1 inhibitor initiation, as indicated in the serial CT scans performed 6 weeks before baseline, and 2 weeks later (Fig. 1).

Previous studies consistently suggested that HPD always resulted in dramatically reduced survival duration, and it limited the opportunity to administer other therapies.^[9–16]^ Fortunately, our patient was diagnosed early via biopsy and treated in a timely manner using CT-guided ^125^I radioactive particle implantation, which was essential for symptom relief and prolonged survival. In addition, the HPD in our case presented with oligoprogression, which was crucial in maximizing the benefit from salvage local therapy. The docetaxel used with the PD1 inhibitor in our case did not thwart HPD. It reminds us that a close follow-up schedule should be established for patients receiving ICI-based combination therapy.

In this report, we described a case of lung cancer showing rapid progression of rib metastasis soon after ICI-based combination therapy. To the best of our knowledge, this is the first case in which HPD was successfully treated with ^125^I radioactive particle implantation. Early detection and adequate treatment are essential for prolonged survival. Further studies are needed to elucidate the molecular and immunological bases of HPD to improve the management of patients receiving ICI therapy.

### 3.1 Consent

Written informed consent was obtained from the patient for publication of this case report and any accompanying images. A copy of the written consent is available for review by the Editor-in-Chief of this journal.

## Author contributions

**Writing – original draft:** Zengjun Liu.

**Writing – review & editing:** Ning Yang, Pinliang Zhang, Zengjun Liu.
